# The value of ultrasound guided laser ablation in papillary thyroid recurrence carcinoma: A retrospective, single center study from China

**DOI:** 10.3389/fendo.2022.946966

**Published:** 2022-09-09

**Authors:** Liang Yong-ping, Zhang Juan, Jing-wu Li, Huai-hua Qi, Jing-ping Liu, Yong-feng Zhao, Wen-gang Liu, Xing-hao Zhang, Ping Zhou

**Affiliations:** ^1^ Department of Ultrasound, Tangshan People’s Hospital, North China University of Science and Technology, Tang Shan, China; ^2^ Department of Breast Surgery, Tangshan People’s Hospital, North China University of Science and Technology, Tang Shan, China; ^3^ Department of Ultrasound, The Third Xiangya Hospital, Central South University, Changsha, China

**Keywords:** thyroid cancer, recurrent, percutaneous thermal ablation, laser, minimally invasive treatment, ultrasound

## Abstract

**Objective:**

The efficacy and safety of ultrasound-guided percutaneous laser ablation (PLA) for treating recurrent papillary thyroid cancer nodules (RPTCNs).

**Methods:**

A retrospective study was conducted in 43 patients with single recurrent thyroid cancer which was diagnosed by fine needle aspiration biopsy (FNAB). The extent of ablation was assessed by contrast-enhanced ultrasound (CEUS) 24h after PLA. At baseline (before ablation), 6, and 12 months, and every 6 months thereafter, the following were recorded: nodule maximum diameter, volume reduction rate (VRR), complications, and side effects.

**Result:**

All 43 patients were successfully treated with PLA without serious complications. All patients underwent CEUS 24 hours after PLA treatment, and all achieved complete ablation. The success rate of single ablation was 100%. The average follow-up time was 23.47 ± 6.50 months, 12 ~ 36 months. At the last follow-up, 32 (74.4%) ablation lesions disappeared completely and 11 (25.6%) ablation lesions showed scar-like changes. No lymph node metastasis was found during follow-up. The maximum diameter and volume of nodules decreased from 5.1 ± 1.4 mm, 86.22 ± 20.46 mm^3^ before operation to 0.73 ± 1.1 mm, 1.02 ± 1.92 mm^3^ at the end of observation (*P* < 0.01). The average volume reduction rates (VRR) at 6, 12, 18, 24, 30 and 36 months after ablation were 11.92%, 60.64%, 82.26%, 90.96%, 93.7% and 97.79% respectively. No regrowth of treated nodule and distant metastases were detected. One patient (2.3%) had local recurrence and was treated with PLA again.

**Conclusion:**

Ultrasound-guided PLA appears to be effective and safe for treating unifocal RPTCNs in selected patients who are ineligible for surgery, which is suitable for clinical application and promotion.

## Introduction

Thyroid nodules are commonly encountered in the clinical practice, and affect 20-50% of the general population as shown by ultrasound (1). Some of these thyroid nodules (14%) are malignant, confirmed by FNAB (2). The most common pathological type of thyroid cancer is papillary carcinoma, among which papillary thyroid micro carcinoma (PTMC) is the most common subtype (3, 4). Although the incidence rate of PTMC is increasing year by year, the mortality rate is very low (5). The current management of PTMC has become more conservative. The thyroid unilateral lobectomy is the most common treatment adopted by surgeon in PTMC of single lesion (6). Most patients recovered after operative treatment, but the risk of postoperative recurrence is still as high as 30% (7). Thyroid surgery is often accompanied by adhesion of anterior cervical tissue, and the structure is complex and difficult to distinguish, which leads to difficulty in reoperation, thus the complication rate of reoperation is about 5 to 10 times higher than that of the first operation (8). Therefore, it is particularly important to find a new minimally invasive treatment. Ultrasound-guided PLA has developed rapidly in recent years and has been widely used in the treatment of liver, kidney and lung tumors (9, 10). Our previous study has confirmed that PLA has high VRR with good preservation of thyroid function and few complications for thyroid tumor (11). Yet, the experience with PLA in recurrent papillary thyroid cancer nodules (RPTCNs) is limited. To the best of our knowledge, there has been no systematic study that investigated the clinical efficacy and safety of PLA for RPTCNs.

## Methods

### Patient

The Ethics Committee of Third Xiangya Hospital of Central South University approved this retrospective study and waived the requirement of written informed consent. Each patient provided written informed consent before PLA after full explanation of the purpose and nature of the procedure used. The ablations were performed in accordance with approved guidelines and regulations, in a dedicated interventional operating room by two doctors who both had more than 10 years’ experience in this procedure performed the PLAs.

For this study, all the patients conformed to the following inclusion criteria (**Table 1**): a. cytological diagnosis showing PTMC in patients with history of thyroid unilateral lobectomy due to papillary thyroid cancer, b. single lesion with a maximum diameter less than 10mm, c. a minimum distance from the lesion to the thyroid capsule of ≥3mm, d. no tumor invasion to the extra-thyroid organs (trachea, common carotid artery or esophagus), e. patients who were not eligible for surgery or unwilling to undergo surgery due to high surgical risk or other reasons, and f. patients who were anxious and affected their normal life or unwilling to undergo clinical observation. Patients with any of the following were excluded from this study (**Table 1**): a. cytological diagnosis showing another type of thyroid malignancy such as medullary carcinoma, b. clinically apparent multi-centricity, c. other nodules with sonographic features suggestive of malignancy including microcalcification, local invasion, height greater than width, and markedly reduced echogenicity, d. the lesions were close to trachea, esophagus or common carotid artery, and hydrodissection was difficult to establish, e. lesions located in the isthmus, f. ultrasound or other image examination revealing suspicious cervical or distant lymph node metastasis, and g. unable to cooperate with the puncture.

**Table 1:** Inclusion and exclusion criteria in this study.

Item	Details
Inclusion criteria	1. cytological diagnosis showing PTMC in patients with history of thyroid unilateral lobectomy due to papillary thyroid cancer
	2. single lesion with a maximum diameter less than 10mm
	3. a minimum distance from the lesion to the thyroid capsule of ≥3mm
	4. no tumor invasion to the extra-thyroid organs (trachea, common carotid artery or esophagus)
	5. patients who were not eligible for surgery or unwilling to undergo surgery due to high surgical risk or other reasons
	6. patients who were anxious and affected their normal life or unwilling to undergo clinical observation
Excluded criteria	1. cytological diagnosis showing another type of thyroid malignancy such as medullary carcinoma
	2. clinically apparent multi-centricity
	3. other nodules with sonographic features suggestive of malignancy including microcalcification, local invasion, height greater than width, and markedly reduced echogenicity
	4. the lesions were close to trachea, esophagus or common carotid artery, and hydrodissection was difficult to establish
	5. lesions located in the isthmus
	6. ultrasound or other image examination revealing suspicious cervical or distant lymph node metastasis
	7. unable to cooperate with the puncture

### Equipment

The ultrasound system was a MyLab Twice color Doppler ultrasound system (Esaote, Italy) equipped with contrast-enhanced ultrasound imaging technology. High-resolution linear probes (6-12 MHz) were used to monitor and pre-ablation assessment, ablation therapy, and follow-up.

PLA was conducted with an ultrasonic laser integrated system produced by Italian Esaote Medical, and Echolaser X4 laser (Nd : YAG laser) treatment system. The device comprised a 1,064-μm diode laser unit with a maximum of 4 laser sources, each with an individual energy emission setting and independent activation, 0.3-mm diameter optic fiber, 21-gauge Chiba needle, and a foot pedal.

### Pre-ablation evaluations

To determine the diagnosis of RPTCNs before ablation, a cytologic examination by fine-needle aspiration biopsy was necessary. The ultrasound examination was indispensable for identifying and classifying the target nodule and characterizing the anatomical relationship between the nodule and the important surrounding structures. The maximum diameter and two orthogonal diameters, echoic characteristics, internal blood flow distribution, and the ratio of solid components of each nodule were obtained by an experienced sonographer. The nodule volume (V) was estimated by the ellipsoid formula V = π×a×b×c/6, where a is the maximum diameter, and b and c are the 2 orthogonal diameters.

Laboratory tests were conducted prior to ablation, consisting of a complete blood count and blood coagulation, and the following thyroid function indicators: thyroid stimulating hormone, triiodothyronine (T3), free thyroxine (FT4), and calcitonin.

### Ablation procedure

**Figure 1:**
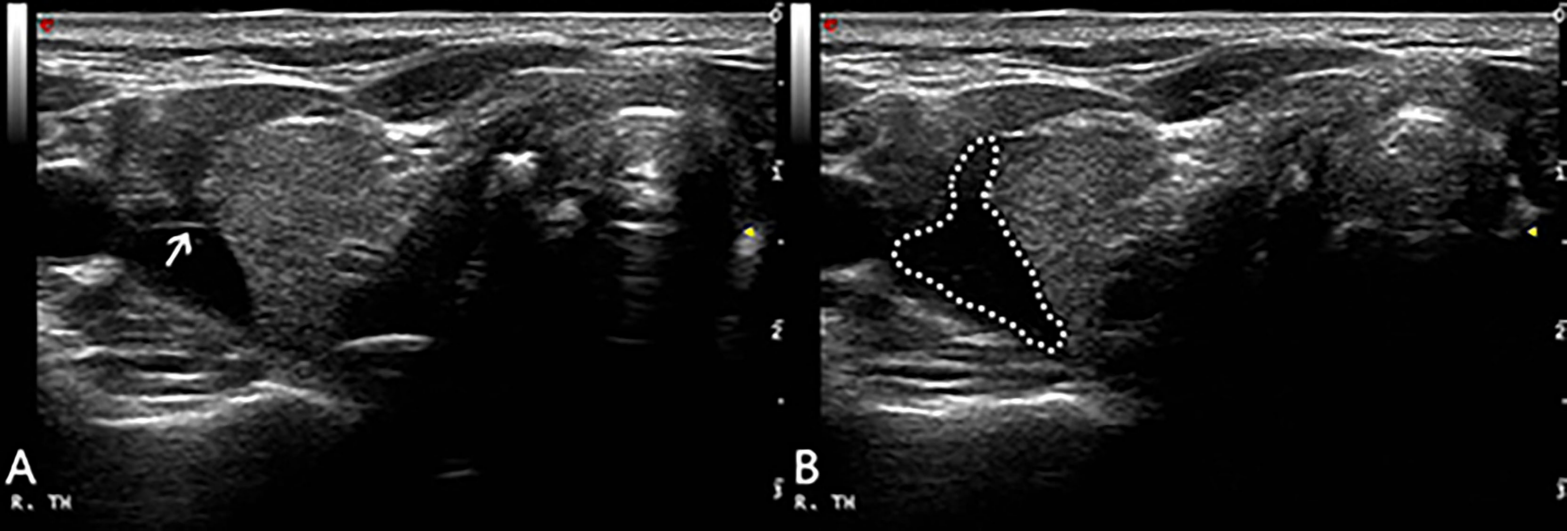
After local anesthesia, a mixture of 2% lidocaine and physiological saline was injected into the surrounding thyroid capsule by 23-gauge needle (arrow in **A**), achieving a ‘hydrodissection’ (dotted line in **B**) to protect the common carotid artery from thermal injury.

The patient was supine with the neck fully exposed. The target nodule location and its adjacent structures were evaluated under ultrasound and the puncture route was pre-designed. All procedures were performed under aseptic conditions and local anesthesia with 1% lidocaine. In order to protect the vital organs (trachea, recurrent laryngeal nerve, common carotid artery and esophagus) from thermal injury, a bolus of 2% lidocaine and physiological saline solution (1:8 dilution) was carefully infused into the surrounding thyroid capsule to achieve a hydrodissection (12, 13), the width of which was not less than 5mm (**Figure 1**).

**Figure 2:**
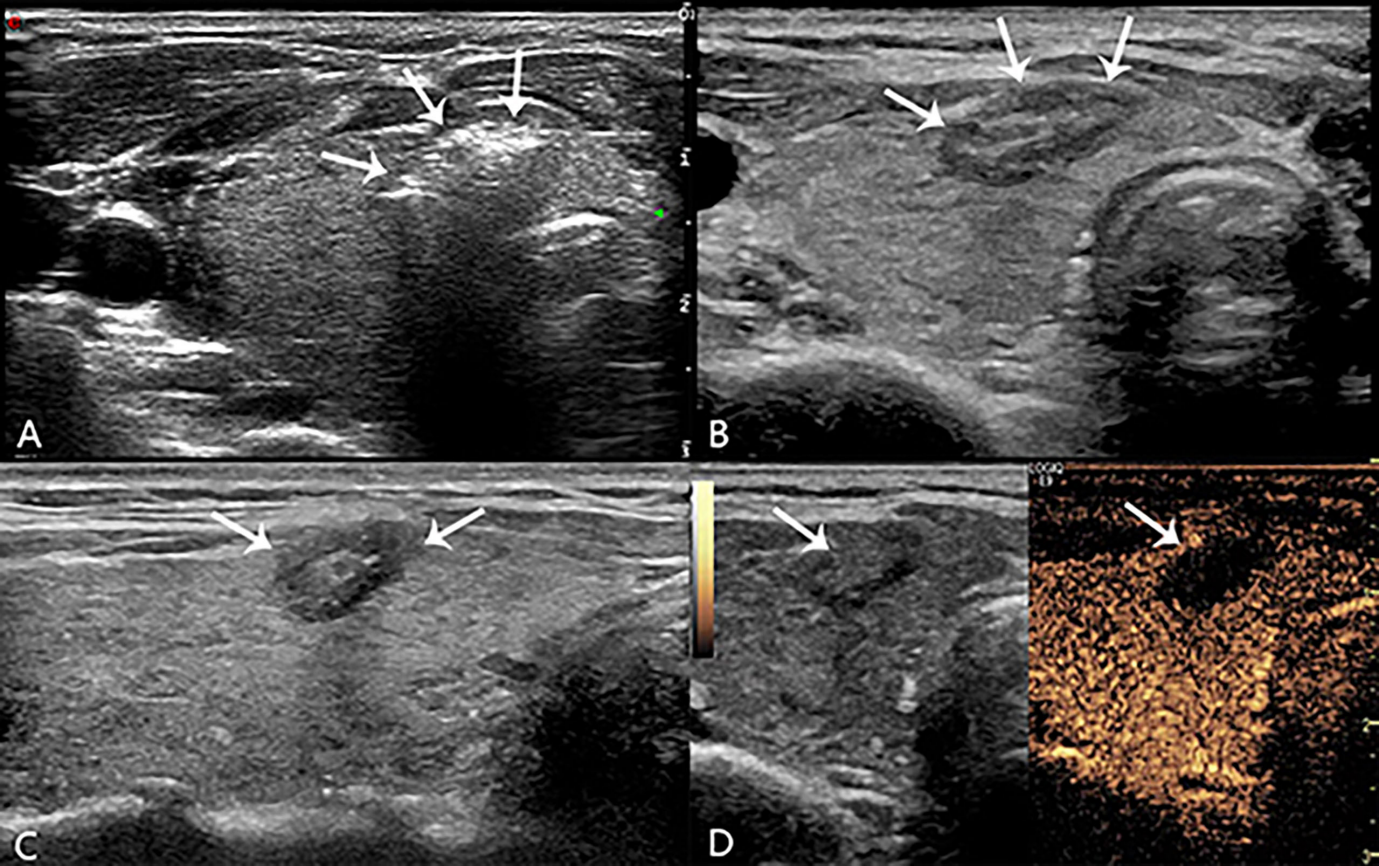
During laser lation the hyperechoic area (arrow in **A**) around the pointed end of the optical fiber. The lesion (arrow in **B**, transverse plane; arrow in **C**, longitudinal plane) appeared medium-high echogenicity in the ablation area and hypoecho in the surrounding area, with a clear boundary between the ablation area and normal tissue 24h after operation. There was no contrast agent perfusion in the ablation area (arrow in **D**) after PLA 24h on CEUS.

During the injection, the patient was questioned for short answers to monitor the status of phonation. After anesthesia, the 21-gauge guide needle was percutaneously penetrated into the center of the target lesion under realtime ultrasound guidance, and then the core needle was pulled out. Then, a plane-cut optic fibre was inserted through the sheath of the 21-gauge needle into the same position. The output power of the laser instrument was 3W with the initial energy 1200 joule. The specific energy changes according to the size of the nodule. After laser activation, a hyperechoic area was gradually formed and expanded around the pointed end of the optical fiber on sonograph. When the hyperechoic area completely covered the lesion and the surrounding area of about 3mm, then the needle was removed while cauterizing the needle path with 3W until the energy output was stopped outside the thyroid capsule and the needle was pulled out. Sterile application was applied to the puncture point. Meanwhile, the doctor or nurse compressed the patient’s neck for 30 min to avoid bleeding. The ablation time and total energy were recorded, and immediately evaluate whether there are complications such as pain (Visual Analogue Scale/Score, VAS) and hoarseness. Routine ultrasound and CEUS (SonoVue, Bracco, Milan, Italy) were performed 24 h after operation to evaluate whether the ablation was complete (**Figure 2**), the patients were observed in the hospital during this time. If there were no signs of complications and the patient’s vital signs were normal, they were allowed to leave the hospital after 24 h.

### Post-ablation assessment

The success of ablation was assessed afterward *via* CEUS, and included the ablative range, nodule echo, blood flow, and non-ablated portion. At postoperative 3, 6, and 12 months during the first year of follow-up, and every 6 months thereafter, the following were noted: the diameter and volume of the ablated nodules and laboratory tests results such as T3, FT4 and thyroid stimulating hormone. The volume reduction rate (VRR) was the percentage change in volume after ablation. The VRR was calculated as a percentage as follows: (initial volume - final volume) × 100/initial volume. If suspicious lymph nodes or lesions in thyroid were found, ultrasound-guided FNAB was performed.

### Statistical analysis

Data analyses were performed using SPSS statistical software version 22.0 (SPSS, Chicago, IL). Categorical variables are expressed as frequencies, and continuous variables as mean ± standard deviation. Self-paired T test was used to compare the volume of thyroid nodules before and after ablation. A *P* value < 0.05 was considered statistically significant.

## Results

### Patient

From September 2017 to September 2020, 43 patients conformed to the inclusion criteria for this study (**Table 1**). All patients enrolled were given unilateral lobectomy because of papillary thyroid cancer in other hospitals before. In order not to undergo another surgery they chose to join in this study. The initial maximum diameters and volumes of the nodules were 5.1 ± 1.4 mm and 86.22 ± 20.46 mm^3^.

### Ablation procedure

Among the total 43 nodules treated, hydrodissection was performed in 39 (90.7%) treatments. The energy and ablative time of ablation applied overall were 1185.02 ± 210.42 joule and 395.01 ± 70.14 s (**Table 2**).

**Table 2:** Baseline characteristics of patients with RPTCNs treated with PLA.

Item	Number
Subjects	43
Female, n (%)	31 (72)
Age, year	38.72 ± 11.13
Age at diagnosis, year	31.74 ± 9.97
Time between primary and recurrence, year	7.58 ± 2.06
Diameter, mm	5.1 ± 1.4
Volume, mm^3^	46.22 ± 20.46
Total energy, J	1185.02 ± 210.42 (840-1500)
Total ablation time, s	395.01 ± 70.14 (280.0~500)
VAS	2.8 ± 1.3
FU period, mo (%)	36(11) 30(29) 24(54) 18(79) 12(100)

Values are reported as mean ± SD. FU, follow-up; mo, months; J, Joule.

### Hematological index

Before and after the ablations, each patient’s thyroid laboratory tests results including thyroid stimulating hormone, T3, T4, FT3 and FT4, and blood routine including WBC and RBC were tested. All the results were within normal range, proving the safety and protection of the thyroid function of the ablations (**Table 3**).

**Table 3:** Thyroid laboratory tests and blood tests results in pre- and post-ablation.

Time	TSH(μIU/ml)	T3(ng/ml)	T4(μg/dl)	FT3(pg/ml)	FT4(ng/dl)	WBC(10^9/L)	RBC(10^12/L)
Pre	0.48 ± 0.26	0.87 ± 0.41	5.73 ± 1.03	2.02 ± 0.48	0.91 ± 0.14	7.28 ± 1.10	4.72 ± 0.69
1 mo	0.49 ± 0.32	0.92 ± 0.50	6.20 ± 1.27	2.23 ± 0.54	1.13 ± 0.21	7.79 ± 1.35	4.66 ± 0.85
3 mo	0.47 ± 0.30	0.90 ± 0.47	6.07 ± 1.01	2.16 ± 0.51	1.05 ± 0.17	7.64 ± 1.21	4.81 ± 0.53
6 mo	0.46 ± 0.24	0.88 ± 0.49	6.19 ± 1.17	2.07 ± 0.47	1.01 ± 0.11	7.38 ± 1.24	4.77 ± 0.76
12mo	0.47 ± 0.29	0.86 ± 0.44	6.31 ± 1.13	2.14 ± 0.56	0.98 ± 0.15	7.04 ± 1.06	4.61 ± 0.62

Values are reported as mean ± SD. Pre, pre-ablation; mo, months.

### Follow-up evaluation

**Figure 3:**
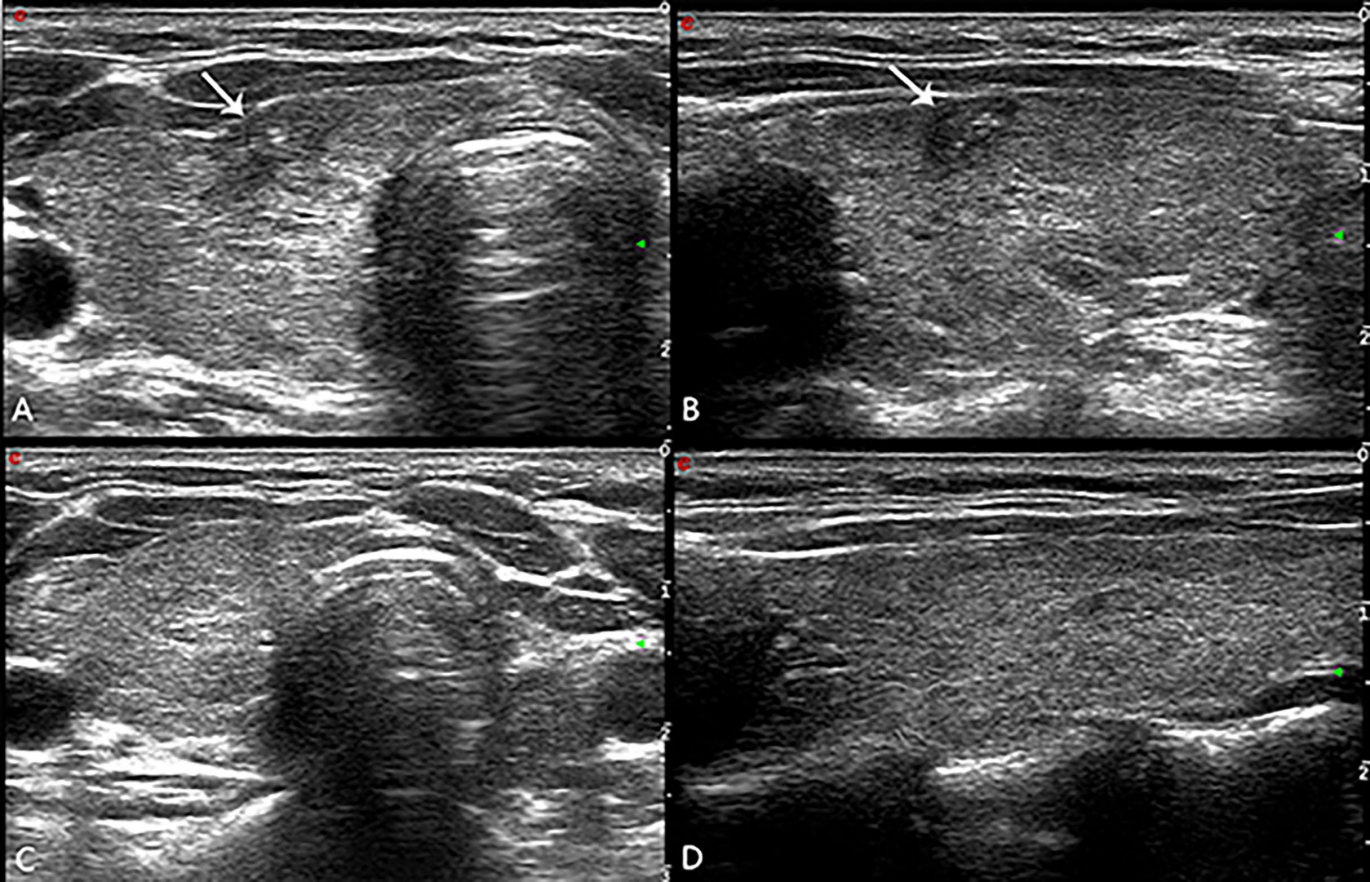
**(A)** The ablation lesion (arrow in **A**, transverse plane; arrow in **B**, longitudinal plane) shrank gradually after PLA 3 months. The ablation lesion (arrow in **C**, transverse plane; arrow in **D**, longitudinal plane) disappeared completely after PLA 30 months.

Ultrasound showed medium-high echogenicity in the ablation area and hypoecho in the surrounding area, with a clear boundary between the ablation area and normal tissue 24h after operation. Color Doppler ultrasound showed absence of blood flow in the ablation area. CEUS showed that there was no contrast agent perfusion in the ablation area in all 43 patients, and the non-perfusion area was completely covered and exceeded the original tumor area, which suggested complete ablation, and the success rate of single ablation was 100%. The volumes of the ablated nodules at 6, 12, 18, 24, 30 and 36 months were 40.71 ± 60.59, 18.19 ± 28.52, 8.2 ± 13.52, 4.18 ± 7.73, 2.91 ± 4.39 and 1.02 ± 1.92 mm^3^ respectively, which were much less than that before ablation (*P* < 0.05, all). The longest follow-up time was 36 months, and the average follow-up time was 23.47 ± 6.50 months. The volume of all ablation lesions was gradually reduced. The mean maximum diameter and mean volume of ablation decreased from 5.1 ± 1.4 mm and 46.22 ± 20.46 mm^3^ before ablation to 0.73 ± 1.1 mm and 1.02 ± 1.92 mm^3^ (*P* < 0.01), respectively. The average of VRR was 97.79%, 32 (74.4%) ablation lesion disappeared completely, and 11 (25.6%) ablation lesion showed scar-like changes (**Figure 3**). The average of VRR at 6, 12, 18, 24, 30 and 36 months after laser ablation were 11.92%, 60.64%, 82.26%, 90.96%, 93.7% and 97.79% respectively (**Table 4**)

**Table 4:** Outcomes of RPTCNs treated after PLA.

Time	N	Max D, mm		Volume, mm^3^		VRR (%)	*P*
		Mean	scale	Mean	Scale		
BL	43	5.1 ± 1.4	3.5-8.1	46.22 ± 20.46	18.41-179.80	–	–
1D	43	17.29 ± 4.1	9.96-26.8	618.6 ± 503.90	110.7-1802.5	-1238.38	0.00
1M	43	13.97 ± 3.73	7.72-21.03	313.13 ± 270.74	52.63-1202.94	-577.48	0.00
3M	43	9.91 ± 3.18	3.93-16.18	119.30 ± 116.25	7.39-484.83	-158.11	0.00
6M	43	6.87 ± 2.73	1.03-13.54	40.71 ± 60.59	0-302.16	11.92	0.00
12M	43	3.30 ± 2.41	0.7-10.2	18.19 ± 28.52	0-157.13	60.64	0.00
18M	35	1.99 ± 2.23	0-7.4	8.2 ± 13.52	0-74.7	82.26	0.00
24M	24	1.14 ± 1.6	0-4.1	4.18 ± 7.73	0-34.76	90.96	0.00
30M	13	1.0 ± 1.42	0-3.6	2.91 ± 4.39	0-30.24	93.7	0.00
36M	5	0.73 ± 1.1	0-2.8	1.02 ± 1.92	0-24.22	97.79	0.00

BL, baseline; D, diameter; VRR, volume reduction rate.

Thyroid parenchyma and lymph nodes: during the whole follow-up period, only one (2.3%) patient was found. At the follow-up of 12 months after operation, a 4-mm-sized tumor was found in another part of the gland, which was confirmed as PTMC by FNAB. Imaging examination showed no obvious lymph node metastasis. Laser treatment was performed again for the request of the patient. The patient was followed up for 2 years at the end of the study, and there was no local recurrence and lymph node metastasis. The recurrence free survival rate within 3 years was 97.68%. During the follow-up period, no patient had lymph node metastasis, and the metastasis free survival rate was 100%.

### Safety assessment

There were 40 (93%) major complications in this study, most of which were mild. Of those who experienced pain, the pain scores were 2.8 ± 1.3. Three (7%) patients had the minor complication of pain in the ablation area or radiated to the head, ears, shoulders, or teeth, especially when the ablated lesion was close to the anterior cervical muscles which disappeared within two weeks without any sequelae, no patient needed to stop the ablation due to intolerance. According to the previous doctor’s order, thyroid function of all patients were at normal level before operation. Thyroid function was reexamined one month after operation, 2 (4.65%) patients had thyroid dysfunction transiently, and one patient had a slight decrease in TSH, which returned to normal two months after operation without any additional drug treatment, and another patient had an increase in thyroglobulin antibody, which returned to normal half a year of follow-up. All patients had no serious complications such as trachea, esophagus, vascular injury or tissue swelling and skin scald at the ablation site.

## Discussion

The biological behavior of RPTCNs is mild with very low mortality (14). At present, the clinical treatment of patients with recurrent papillary thyroid cancer includes surgical treatment and clinical follow-up (15). Preliminary studies suggested that the cervical tissue adhesion caused by the first operation will significantly increase the difficulty of the second operation and postoperative complications, such as recurrent laryngeal nerve injury, hypocalcemia (16, 17). Due to poor basic condition some patients were intolerant to the surgical operation, other patients worried about that operation may induced cosmetic problems, psychological resistance and other problems (18). Furthermore. Some patients are reluctant to accept the clinical follow-up plan, which leads to anxiety and panic. Ultrasound-guided PLA is minimally invasive and common treatments for patients with thyroid nodules. A preliminary clinical study with a short-term follow-up showed that PLA is a clinically effective, repeatable, and efficient outpatient treatment that is well-tolerated and is associated with a low risk of major complications (19). In addition, recent studies suggested that PLA also shows good safety and efficacy in the treatment of T1N0M0 thyroid cancer (20, 21). Compared with radiofrequency ablation and microwave ablation, PLA has its unique advantages in the treatment of thyroid diseases which adjacent to important organs and tissues, because its laser fiber is tenuous and can be punctured with 21-gauge needle. Besides that, the output energy of PLA is accurate and controllable (22). However, the experience of PLA in the treatment of recurrent PTMC is still in the exploratory stage.

In our study, 43 cases of RPTCNs treated with PLA were followed up for a long time. The longest follow-up time was 3 years, with an average time of 23.47 ± 6.50 months. The success rate of first-time ablation in all patients was 100%. These findings are consistent with previous studies of PLA. Zhou W et al. (23) directed the success rate of first-time ablation as 99.9%. Yue Wenwen et al. (24) and Pacella CM et al. (19) both reported that the success rate of first-time ablation with microwave ablation and PLA were both 100%. After the operation, we analyzed that the possible reason for the high ablation success rate is that the 21-gauge Chiba needle can be more accurately placed in the center of the nodule before ablation. The gasification area not only covered the lesion and surrounding normal glands, but also reaching more than 1000J on total ablation energy. To our delight, the thyroid and surrounding tissues had not appeared severely thermal damage after the operation.

At the end of follow-up, 74.4% (32/43) ablation lesions disappeared completely and 25.6% (11/43) ablation lesions showed scar-like changes. Zhang et al. (25) also reported that the VRR rate of the tumor was close to 100% after radiofrequency ablation. This is probably due to more heat sources in PLA, the temperature of which is higher compared with radiofrequency ablation, and the ablation bias is to ensure that the total energy is sufficient to inactivate the lesion, thus tissue carbonization is more and complete disappearance is less likely. The local curative effect was definite and there was no lymph node metastasis, which proved that the late curative effect of PLA was worthy of affirmation. During the follow-up period, one patient had local recurrence (the recurrence rate was 2.3%), and there was no case of lymph node metastasis. The case of local recurrence may be related to the multifocal character of differentiated thyroid cancer, and may also be related to the highly invasive subtype of thyroid papillary carcinoma. Wenwu Dong et al. (26) reported that the recurrence rate of PTC was up to 3.6% after operation in the operated thyroid bed or contralateral residual thyroid tissue. While, the energy of laser ablation is relatively concentrated, the boundary between the ablation edge and the surrounding normal tissue is clear, and the diffusion to the surrounding gland is less. The patient with recurrence underwent ablation again and was followed up for 3 years. There was no local recurrence and lymph node metastasis.

The complications/side effects of PLA treatment were less. There were no serious complications in this study. The reason may be related to the “liquid isolation method” adopted during the ablation of nodules, which adjacent to important tissues and organs, and accurate control with low-power (3W) of laser ablation. In our study, one patient was found that serum thyrotropin decreased, and another patient was found thyroglobulin antibody increased one month after operation. What’s more, two patients were found serum thyrotropin decreased and serum thyroxine increased at one month follow-up. In the later stage, the biochemical indexes of the two patients gradually returned to normal without any clinical treatment. The temporary abnormality of thyroid function after PLA may be related to the edema of thyroid parenchyma and the thermal injury of thyroid capsule after ablation. This might indicate that thyroid tissue damage caused by PLA is transient and self-limiting. During the ablation process, patients responded to different degrees of pain. Most of the cases (40/43) had mild pain and a few (3/43) patients had moderate pain. Only one patient complained of severe pain half hour after operation and got relieved after using ice to reduce the swelling. Zhao et al. (27) also reported that most patients have neck pain and burning during operation, which may be related to the location of nodules and the pain threshold of patients.

There are still some deficiencies in this study. Firstly, ultrasonography is less sensitive to cervical lymph nodes, especially metastatic lymph nodes in area VI. The possibility of small metastasis cannot be ruled out in cases with negative ultrasonography. Secondly, the first surgical pathological results and subtypes of some patients in this study are not completely clear, and the prognosis of different subtypes is not exactly the same. Finally, this study is a self-control retrospective study. In the future, large samples and multi centers are needed for further prospective controlled research.

## Conclusion

Ultrasound-guided PLA appears to be effective and safe for treating unifocal RPTCNs in selected patients who are ineligible for surgery, which is suitable for clinical application and promotion.

## Data availability statement

The raw data supporting the conclusions of this article will be made available by the authors, without undue reservation.

## Ethics statement

The studies involving human participants were reviewed and approved by The Ethics Committee of Third Xiangya Hospital. The patients/participants provided their written informed consent to participate in this study.

## Author contributions

L-YP and ZJ have equal contributions to this article. LY-P: conceptualization and writing-original draft. ZJ: formal analysis and project administration. PZ: writing-review and editing. Y-FZ: resources. W-GL: supervision. X-HZ: investigation. J-WL: software. H-HQ: visualization. J-PL: validation. All authors contributed to the article and approved the submitted version.

## Funding

This work was supported by grants from the National Natural Science Foundation of China (No.81871367), Project of Hebei Provincial Health Commission (No. 20181231), Project of Hunan Provincial Health Commission (No. 202209025123) and the Natural Science Foundation of Hunan Province, China (No. 2021JJ31037).

## Conflict of interest

The authors declare that the research was conducted in the absence of any commercial or financial relationships that could be construed as a potential conflict of interest.

## Publisher’s note

All claims expressed in this article are solely those of the authors and do not necessarily represent those of their affiliated organizations, or those of the publisher, the editors and the reviewers. Any product that may be evaluated in this article, or claim that may be made by its manufacturer, is not guaranteed or endorsed by the publisher.

## Abbreviations

RPTCNs, Recurrent papillary thyroid cancer nodules; PTMC, Papillary thyroid micro carcinoma; CEUS, Contrast-enhanced ultrasound; FNAB, Fine needle aspiration biopsy; PLA, Percutaneous laser ablation; VRR, Volume reduction rates.
